# Different Types of White Matter Hyperintensities in CADASIL

**DOI:** 10.3389/fneur.2018.00526

**Published:** 2018-07-10

**Authors:** Edouard Duchesnay, Fouad Hadj Selem, François De Guio, Mathieu Dubois, Jean-François Mangin, Marco Duering, Stefan Ropele, Reinhold Schmidt, Martin Dichgans, Hugues Chabriat, Eric Jouvent

**Affiliations:** ^1^NeuroSpin, CEA, Université Paris-Saclay, Gif-sur-Yvette, France; ^2^Institute for Energy Transition (ITE), VEDECOM, Versailles, France; ^3^UMR-S 1161 INSERM, Université Paris Diderot, Sorbonne Paris Cité, Paris, France; ^4^Institute for Stroke and Dementia Research (ISD), University Hospital, LMU Munich, Munich, Germany; ^5^Department of Neurology, Medical University of Graz, Graz, Austria; ^6^Department of Neurology, Lariboisière Hospital, Assistance Publique-Hôpitaux de Paris (AP-HP), Paris, France; ^7^DHU NeuroVasc - Sorbonne Paris Cité, Paris, France

**Keywords:** cerebral small vessel disease, white matter changes, white matter hyperintensities, CADASIL, clinical severity

## Abstract

**Objective:** In CADASIL (Cerebral Autosomal Dominant Arteriopathy with Subcortical Infarcts and Leukoencephalopathy), white matter hyperintensities (WMH) are considered to result from hypoperfusion. We hypothesized that in fact the burden of WMH results from the combination of several regional populations of WMH with different mechanisms and clinical consequences.

**Methods:** To identify regional WMH populations, we used a 4-step approach. First, we used an unsupervised principal component algorithm to determine, without a priori knowledge, the main sources of variation of the global spatial pattern of WMH. Thereafter, to determine whether these sources are likely to include relevant information regarding regional populations of WMH, we tested their relationships with: (1) MRI markers of the disease; (2) the clinical severity assessed by the Mattis Dementia Rating scale (MDRS) (cognitive outcome) and the modified Rankin's score (disability outcome). Finally, through careful interpretation of all the results, we tried to identify different regional populations of WMH.

**Results:** The unsupervised principal component algorithm identified 3 main sources of variation of the global spatial pattern of WMH, which showed significant and sometime inverse relationships with MRI markers and clinical scores. The models predicting clinical severity based on these sources outperformed those evaluating WMH by their volume (MDRS, coefficient of determination of 39.0 vs. 35.3%, *p* = 0.01; modified Rankin's score, 43.7 vs. 38.1%, *p* = 0.001). By carefully interpreting the visual aspect of these sources as well as their relationships with MRI markers and clinical severity, we found strong arguments supporting the existence of different regional populations of WMH. For instance, in multivariate analyses, larger extents of WMH in anterior temporal poles and superior frontal gyri were associated with better outcomes, while larger extents of WMH in pyramidal tracts were associated with worse outcomes, which could not be explained if WMH in these different areas shared the same mechanisms.

**Conclusion:** The results of the present study support the hypothesis that the whole extent of WMH results from a combination of different regional populations of WMH, some of which are associated, for yet undetermined reasons, with milder forms of the disease.

## Introduction

White matter hyperintensities (WMH) are a hallmark of cerebral small vessel disease (SVD). While it is still widely considered that they result from chronic hypoperfusion, other mechanisms are likely involved (1, 2).

In CADASIL (Cerebral Autosomal Dominant Arteriopathy with Subcortical Infarcts and Leukoencephalopathy), the most frequent monogenic form of SVD, WMH are commonly seen in anterior temporal poles and superior frontal gyri, which are generally spared by WMH in age- and hypertension-related SVD (3). We recently showed that the WMH observed in these areas in CADASIL are characterized by far longer T1 and T2^*^ relaxometry values than WMH observed in the remaining white matter. This large difference, tightly linked to the local water content (4), suggests that WMH in anterior temporal poles and superior frontal gyri might result from different mechanisms than WMH observed in other brain regions.

**Figure 1:**
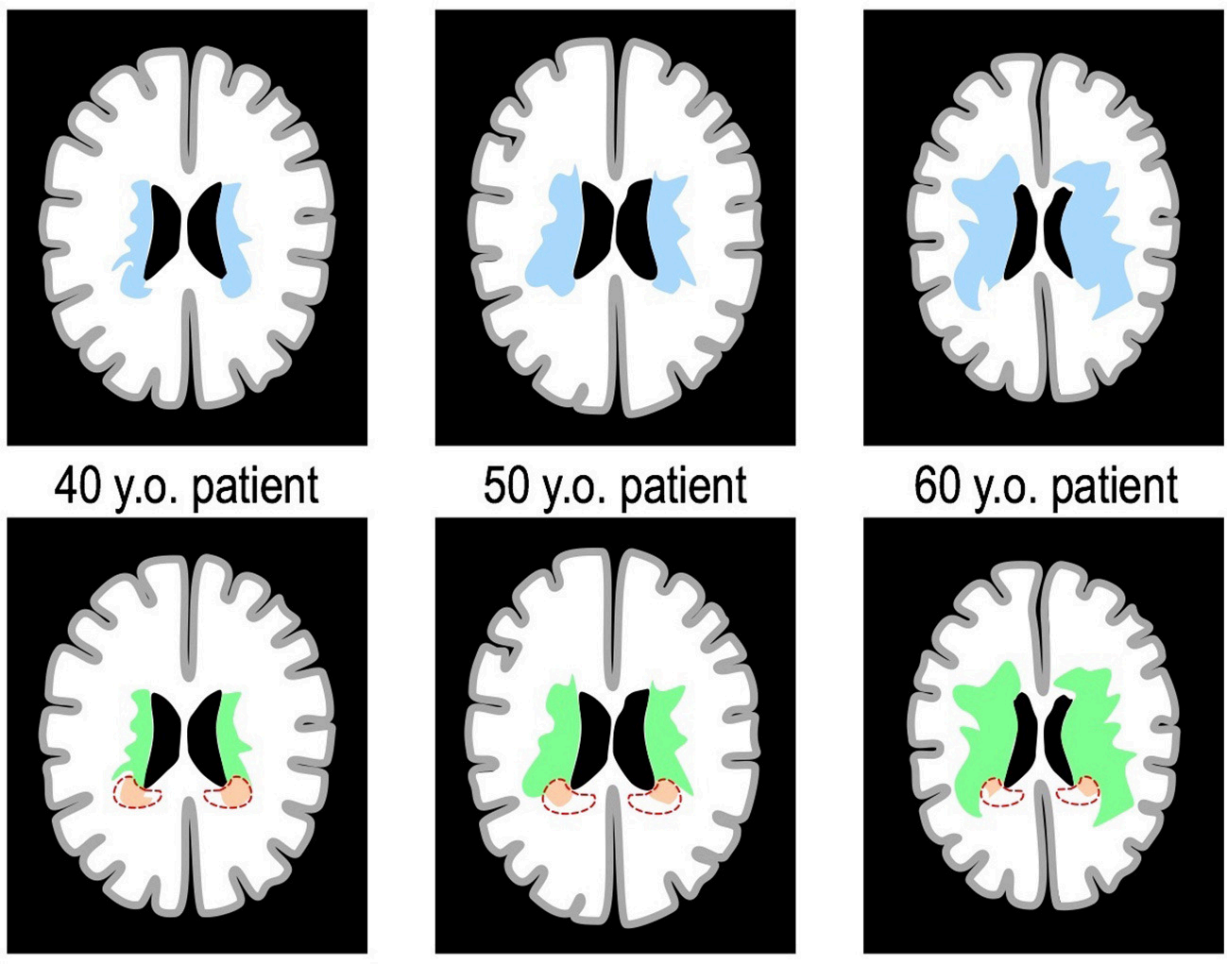
Spatial burden of WMH and the hypothesis of different populations. The schematic represents 3 theoretical patients of increasing age, with on the (**Top Line**) the masks of the whole burden of WMH overlaid on the same axial brain slice (in blue). We hypothesized that the whole burden of WMH results from the combination of different populations of WMH that appear predominantly in certain areas. For instance, the red area on the (**Bottom Line**) represents a theoretical region in which WMH (in orange) would have a specific mechanism inversely related to age. In this case, while the extent of WMH in the red area is inversely related to age, the study of the whole extent of WMH (in blue on the **Top Line**) would not detect this aspect. By contrast, the unsupervised study of the sources of variation of the whole pattern of WMH, used in the present study, would likely detect it.

In the present work, we hypothesized that the whole burden of WMH observed on conventional MRI in CADASIL results from the combination of different regional populations of WMH. To test our hypothesis, we set up a specific imaging analysis protocol to identify, without a priori knowledge, the main sources of variation of the spatial pattern of WMH in a large cohort of CADASIL patients (Figure 1). Thereafter, we aimed to determine, through the careful visual inspection of these sources and the interpretation of their relationships with the other MRI markers of the disease and clinical scores, whether we could identify different regional populations of WMH.

## Materials and methods

### Patients

Three hundred and one consecutive subjects (178 from Paris and 123 from Munich), more than 18 years old, were recruited in a cohort study of CADASIL patients at Lariboisière (Paris) or Ludwig-Maximilians-Universität (Munich) hospitals between October 2003 and April 2009. All participants harboured a typical mutation of the *NOTCH3* gene (5). At inclusion, all patients underwent a thorough neuropsychological and behavioural evaluation (5) as well as a standardized MRI evaluation. For the present study, the 4 main clinical scores reported in previous studies of our group (6–8) were considered for analyses: global cognitive performances were assessed by the Mattis dementia rating scale (MDRS) and mini mental state examination (MMSE); executive functions were assessed by the time to complete part B of Trail Making Test (TMTB), the most sensitive test to executive dysfunction in CADASIL (9); and disability was assessed by the modified Rankin's scale (mRS). A local ethics committee approved the study in both centres.

### MRI

MRI scans were obtained on 1.5-T systems [General Electric Medical Systems Signa (Paris and Munich) or Siemens Magnetom Vision (Munich)]. 3DT1 sequences [Paris: repetition time/echo time (TR/TE) = 9.1/2 ms, slice thickness = 0.8 mm, no interslice gap, in-plane resolution = 1.02 × 1.02 mm^2^; Munich: TR/TE = 11.4/4.4 ms, slice thickness = 1.2 mm, no gap, in-plane resolution = 0.9 × 0.9 mm^2^], FLAIR [Paris: TR/TE/inversion time (TI) = 8402/161/2002 ms, slice thickness = 5.5 mm, no gap, in-plane resolution = 0.94 × 0.94 mm^2^; Munich: TR/TE/TI = 4284/110/1428 ms, slice thickness 5 mm, no gap, in-plane resolution = 0.98 × 0.98 mm^2^] and T2^*^-weighted gradient echo imaging (Paris: TR/TE = 500/15 ms, slice thickness = 5.5 mm, no gap, in-plane resolution = 0.98 × 0.98 mm^2^; Munich: TR/TE = 1056/22 ms, slice thickness = 5 mm, no gap, in-plane resolution = 0.98 × 0.98 mm^2^) were performed. No major hardware or software upgrade was made in either centre during the follow-up period.

### Image processing and analysis

Masks of WMH and of lacunes were semi-automatically determined and the number of microbleeds (MB_N_) recorded in all subjects from FLAIR, 3D-T1 and T2^*^ sequences respectively using validated methods which were detailed previously (10), in agreement with the STRIVE criteria (11). The volumes of WMH and of lacunes (WMH_V_ and LL_V_ respectively) were obtained by multiplying the numbers of voxels in the corresponding masks by the voxel size of the corresponding sequence. Determination of the global brain volume was performed as previously described (12). The brain parenchymal fraction (BPF) was defined as the ratio of brain tissue volume to that of intracranial cavity volume to take into account inter subject variability in head size (13). All masks of WMH were registered to the Montreal Neurological Institute (MNI) template, first with a linear registration between FLAIR and T1 images (FLIRT) and then with a non-linear registration between T1 images and the MNI template (FNIRT) (http://www.fmrib.ox.ac.uk/fsl).

### Statistical methods

For each patient, the whole burden of WMH can be described by the voxels of the white matter in the MNI space corresponding to WMH. This represents for each patient between a few thousands to several hundreds of thousands of voxels. Hence, the comparison of the shapes of WMH between patients, based on information at the voxel level would require billions of computations and yield results at a the scale of the voxel, which would be quite impossible to interpret.

Hence, we used a method to reduce the dimensionality of the statistical problem to a few new variables. To do so, we used a spatially regularized principal component analysis (PCA) approach, adapted to the context of brain images (14), which aim was to identify the main sources (i.e., principal components corresponding to large scale combinations of voxels) that would best describe most of the variations in the shape of WMH among patients. The main principles of the methods are explained in Supplementary Figure 1. The analysis was performed without a priori knowledge of our hypothesis regarding different populations of WMH.

**Figure 2:**
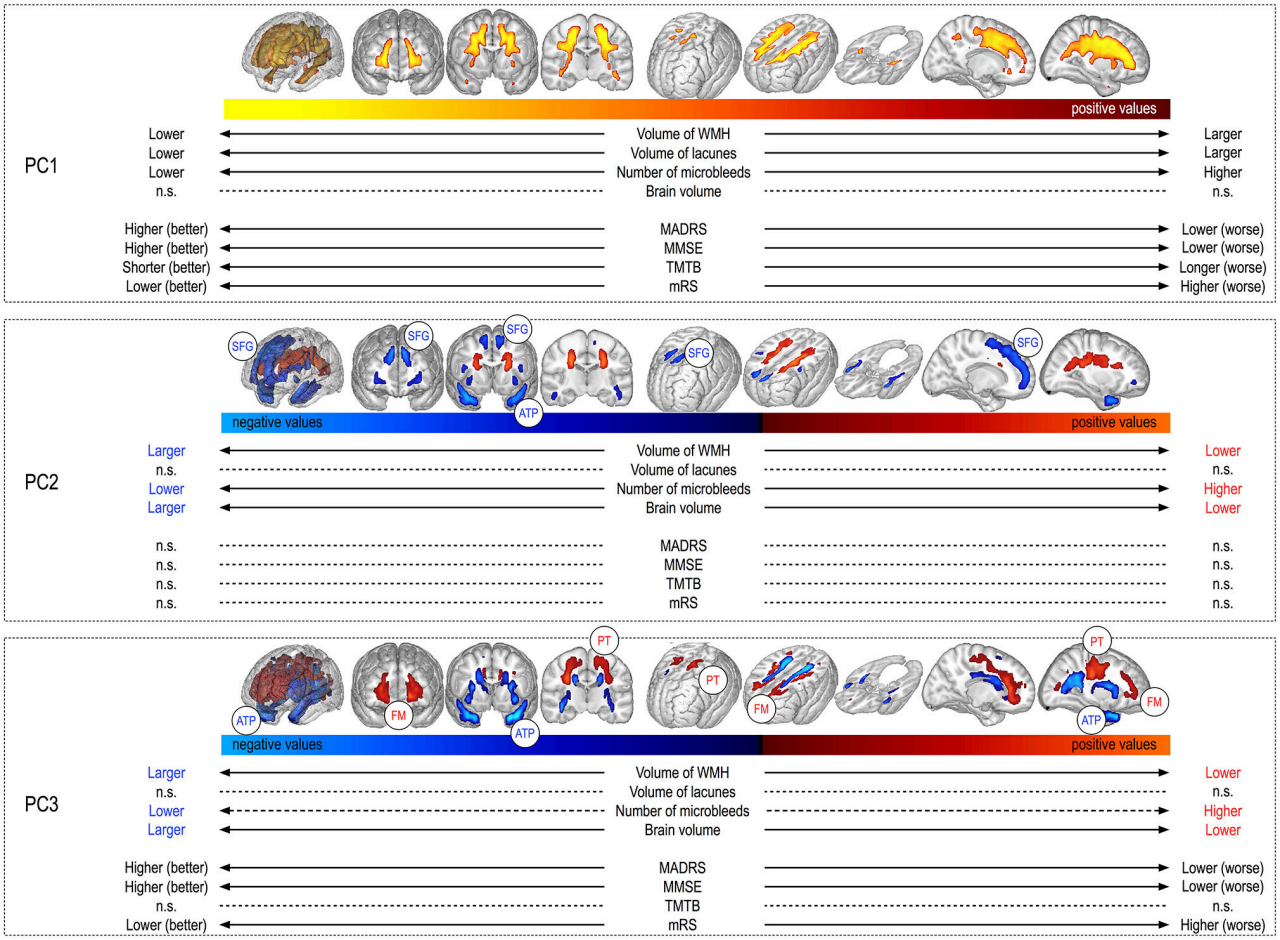
Visual aspect of principal components and their relationships with other MRI markers and clinical scores. Each box depicts one principal component. The **Top** of the box shows the component pattern (the combination of voxels that explains a part of the variability of WMH shape) and the **Bottom** the corresponding component value. The component value describes the position of a given patient with respect to all the others for a given component pattern. For instance, PC1 represents the volume of WMH, and as such, patients with large extents of WMH will have large PC1 values and lie on the right of the colour shade, while those with low extents will lie on the left of the colour shade. The links between the component values and the different MRI markers and clinical scores are also represented. For instance patients with large extents of PC1, having large volumes of WMH, also have larger volumes of lacunes. MDRS, mattis dementia rating scale; MMSE, mini mental state examination; TMTB, trail making test version B; mRS, modified Rankin's Scale; BPF, parenchymal brain fraction; LL_V_, volume of lacunes; WMH_V_, volume of white matter hyperintensities; MB_N_, number of microbleeds; SFG, superior frontal gyrus; ATP, anterior temporal pole; PT, pyramidal tract; n.s., absence of significant relationship between considered variables.

The PCA algorithm performs the analyses in a repeated manner. In turn, the algorithm identifies a principal component (PC) which explains most of the variability in the shape of WMH between patients. Then, the analysis is re-performed on the remaining variability (the initial variability from which is subtracted the variability explained by the last principal components) to identify new components (Figures 1, 2). The algorithm may find a very large number of PC, but the percentage of variability of each PC decreases exponentially. Thus we defined a priori, in line with the usual usage of PCA, a criterion to stop looking for new PC. We chose to stop the addition of new PC as soon as the relative improvement of the total explained variance by the new PC was inferior to 5%.

Each PC is represented by a pattern (a combination of voxels that can be represented visually on 3D meshes, see Figure 2). All the patients are distributed along a given PC through their corresponding PC values. For each PC, the position of a given patient with respect to all the others from the cohort is given by the PC value.

Thereafter, we tested the relationships between the different PC values and: (1) MRI markers of CADASIL, namely BPF, LL_V_, WMH_V_, and MB_N_, with systematic adjustment for age and sex; (2) cognitive scores (MDRS, MMSE, TMTB) and disability scale (mRS), with systematic adjustment for age, sex, level of education, and MRI markers in agreement with the literature in CADASIL (6, 7).

Finally, we compared with ANOVA the models predicting clinical severity based on usual predictors such as age, sex, level of education, BPF, LL_V_ and MB_N_ (reference model) to others further including as a predictor WMH assessed either through their whole volume or through PC values. We calculated for each model the coefficient of determination (*R*^2^), a parameter explaining the part of variance in the outcome explained by a model. We used adjusted *R*^2^ values in order to limit the risk of systematic increase of explained variance with the addition of more predictors.

All statistical analyses were performed with the R software (https://www.r-project.org). Coefficients of determination were calculated with the “relaimpo” package. Multiple imputations were used to deal with missing data (< 2% of outcomes).

## Results

The main clinical and radiological characteristics of the 301 patients are summarized Table 1.

**Table 1:** Clinical and MRI data of the 301 patients.

	**Mean**	***SD***	**Min**	**Max**
Age	50.6	11.2	23	78
Male sex (count - %)	134 (44.5%)
Level of education (count - %)*	0–3: 39 (13%); 4–6: 198 (66%); 7–9: 64 (21%)
MDRS	133.7	17.6	35	144
MMSE	26.9	4.4	6	30
TMTB (time to complete, s)	166.5	164.5	24	975
mRS	0.94	1.29	0	5
BPF (%)	82.4	5.6	62.3	94.6
LL_V_ (mm^3^)	351.7	646.2	0	5180.5
WMH_V_ (mm^3^)	95409.3	66614.9	659.2	414399.9
MB_N_	3.7	13.6	0	141

The clinical and MRI characteristics of the 301 patients of our cohort are summarized. MDRS, mattis dementia rating scale; MMSE, mini mental state examination; TMTB, trail making test version B; mRS, modified Rankin's Scale; BPF, parenchymal brain fraction; LL_V_, volume of lacunes; WMH_V_, volume of white matter hyperintensities; MB_N_, number of microbleeds. *Reference levels of education: 0 = illiterate, 3 = incomplete secondary school (< 9 years), 6 = secondary school (13 years), 7 = university (≥16 years).

### Main sources of variation of the spatial pattern of WMH

The PCA algorithm identified 3 principal components (PC) before reaching the end criterion. The first PC (PC1) explained 19.9% of the variability in the spatial pattern of WMH, further improved by 15% by PC2 and thereafter by 6% by PC3. The patterns associated with the 3 first components are visually represented as masks over whole brain meshes in Figure 2.

The correlation coefficient between PC1 values and WMH_V_ was extremely high (0.95, *p* < 10^−100^), showing that PC1 represents a global quantitative reflect of the whole WMH burden.

Thereafter, PC2 was characterized by inversely related clusters of WMH. Indeed, compared to the pattern predicted in a given patient from the value of PC1, the more negative was the value of PC2, the more WMH voxels were present in blue areas and absent in red areas, and vice-versa (Figure 2).

Finally, PC3 was also characterized by inversely related clusters of WMH voxels. In line, compared to the pattern predicted in a given patient from the values of PC1 and of PC2, the more negative was the value of PC3, the more WMH voxels were present in blue areas and absent from red areas, and vice-versa.

**Figure 3:**
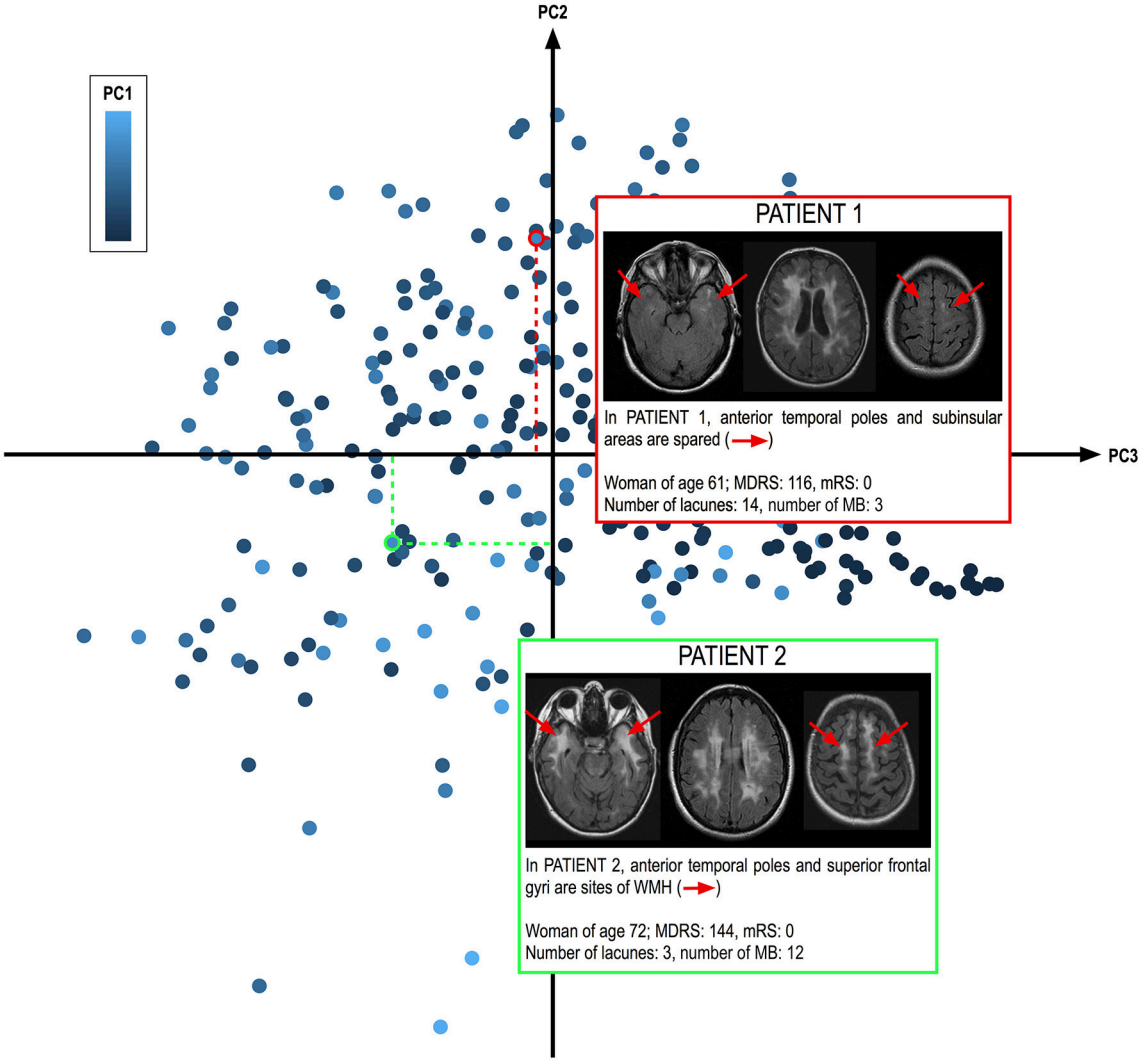
Determination of WMH spatial pattern from the values of the different components. The 301 patients of the cohort are represented according to their PC2 and PC3 values (along the axes) and PC1 score (coded from dark to light blue). Two patients from the cohort with similar PC1 values corresponding to large WMH_V_ (above 140 ml) are shown. These 2 patients clearly illustrate that while their whole extent of WMH is comparable, as illustrated on the middle slice at the level of the centrum semi ovale, their spatial patterns are clearly different. PATIENT 2 shows large extents of WMH in anterior temporal poles and superior frontal gyri, in total contrast with PATIENT 1 who does not. In our cohort, patients with extensive WMH in anterior temporal poles and superior frontal gyri were significantly less severe than the others, independently of known predictors of disease severity, strongly supporting that WMH in anterior temporal poles and superior frontal gyri do not share the same mechanisms that those of other WMH.

A typical illustration of 2 patients with large extents of WMH (corresponding to large PC1 values) but with distinct PC2 and PC3 values are shown Figure 3. While the whole volume of WMH is comparable, the spatial pattern of WMH is totally different between the 2 patients.

### Relationships between the principal component values and other MRI markers (figure 2)

In addition to their strong correlation with WMH_V_, PC1 values were positively and linearly related to LL_V_ (estimate: 2.3 10^−5^, s.e. = 4.3 10^−6^, *p* < 10^−4^) and to MB_N_ (estimate = 4.0 10^−4^, s.e. = 2.1 10^−4^, *p* = 0.04), but not to BPF.

The PC2 values were significantly associated with WMH_V_, BPF, and MB_N_, patients with negative values having larger WMH_V_ (estimate: −3.6 10^−7^, s.e. = 5.0 10^−8^, *p* < 10^−4^), larger BPF (estimate = −5.2 10^−3^, s.e. = 7.1 10^−4^, *p* < 10^−4^), and lower numbers of MB (estimate = 6.9 10^−4^, s.e. = 1.9 10^−4^, *p* = 0.006). By contrast, PC2 values were not significantly related to LL_V_.

In line, PC3 values were also significantly associated with WMH_V_, BPF and MB_N_, patients with negative values having larger WMH_V_ (estimate: −2.8 10^−7^, s.e. = 5.3 10^−8^, *p* < 10^−4^), larger BPF (estimate = −2.9 10^−3^, s.e. = 7.0 10^−4^, *p* < 10^−4^) and lower numbers of MB_N_ (estimate = 5.1 10^−4^, s.e. = 2.0 10^−4^, *p* = 0.05). By contrast, PC3 values were not significantly related to LL_V_.

When considering together the visual aspect of PC2 and PC3 and their relationships with the different MRI markers, it appeared that, independently of potential confounders including the global volume of WMH, the presence of WMH voxels in anterior temporal poles and superior frontal gyri was associated with less severe clinical and MRI phenotypes, while the presence of WMH voxels in pyramidal tracts and forceps minor was associated with, by contrast, more severe forms of the disease (see Supplementary Figure 2).

### Relationships between the 3 principal components and the clinical status

We obtained models with significant abilities to predict the 4 clinical scores based on age, sex, level of education and the different sets of MRI markers (Table 2). In all cases, the adjusted *R*^*2*^ of the corresponding models were high, above 30%. For the 4 clinical scores, the additional variance explained by the inclusion of WMH_V_ as a predictor was weak compared to the models including only BPF, LL_V_, and MB_N_. As expected given the strong correlation observed between PC1 values and WMH_V_, the yield of PC1 values regarding the increase of *R*^2^ was close to that of WMH_V_. Given the strong collinearity between WMH_V_ and PC1 values, we did not tested models including both variables as predictors.

**Table 2:**
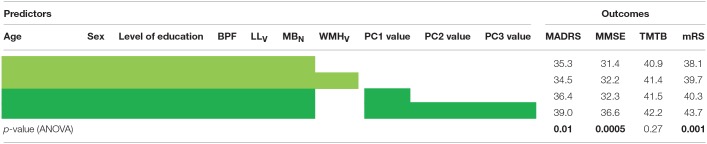
Predictive abilities of the different clinical models evaluated by their coefficients of determination.

*Values of the adjusted coefficients of determination (R^2^) expressed as percentages for the different models predicting the 4 clinical scores. Variables included in the different models are indicated by coloured cells in the left part of the table. Models based on age, sex, level of education, BPF, LL_V_, MB_N_, and PC1 value and models further including PC2 and PC3 values (with corresponding variables in dark green) were compared with ANOVA. P-values corresponding to a significant improvement in the model by further inclusion of PC2 and PC3 values are shown in bold. Please note that given the strong collinearity between PC1 value and WMH_V_ they were not included together in the different models. MDRS, mattis dementia rating scale; MMSE, mini mental state examination; TMTB, trail making test version B; mRS, modified Rankin's Scale; BPF, parenchymal brain fraction; LL_V_, volume of lacunes; MB_N_, number of microbleeds; PC, principal component*.

With the exception of TMTB, all models including PC2 and PC3 values in addition to that of PC1 explained significantly better the clinical outcomes than models including PC1 values or WMH_V_ only (Table 2). The variance explained by the values of PC1, PC2, and PC3 appeared far larger than that of the volume of lacunes in all cases, and close to that of BPF (data not shown).

## Discussion

The results of the present study support the hypothesis that the whole burden of WMH in CADASIL is in fact the combination of different regional populations of WMH, with different mechanisms and clinical consequences.

Indeed, we showed that, independently of the global burden of WMH, the extent of WMH in different brain regions strongly varies among patients. In addition, across different brain regions, we observed different and sometimes inverse relationships between the local extent of WMH and other MRI markers and clinical severity. For instance, all being otherwise equal, larger volumes of WMH in anterior temporal poles and superior frontal gyri are associated with milder forms of the disease, while larger volumes of WMH in pyramidal tracts or in the forceps minor are associated with more severe forms. Finally, the models predicting clinical scores from MRI data performed better when taking into account WMH as a presumed combination of different regional WMH populations rather than when estimating WMH as a whole.

A complex aspect of the present study is the translation between the mathematical objects (the principal components, the sources of variation of the WMH pattern) and the presumably different regional WMH populations. First, it is important to note that our approach is completely different from those testing the local relationships between WMH voxels and the clinical status. These voxel-wise approaches, which allowed demonstrating the importance of alterations in some structures such as the forceps minor in CADASIL (8) and in sporadic SVD (15, 16), indeed rely on the hypothesis that all WMH voxels share the same underlying mechanisms and that their influence on the clinical severity only depends on the structure they are lying on. By contrast, in the present study, we tested the hypothesis that different populations of WMH, with presumably different mechanisms and clinical consequences, systematically appear in certain brain areas.

While our results strongly support the co-existence of different regional WMH population, the interpretation of principal components patterns in terms of regional WMH populations is far from being trivial. Obviously, the blue clusters in PC2 and PC3 in anterior temporal poles and superior frontal gyri matches the pattern of WMH known to be far more frequently observed in CADASIL than in sporadic forms of SVD. Surprisingly, the presence of WMH in these areas was independently associated with milder radiological and clinical phenotypes. In line with recent results showing a specific tissue composition of these WMH when compared to those appearing in other brain areas, our results support not only that these WMH have distinct underlying mechanisms, but also that, for yet unknown reasons, they may be protective against the effects of the disease. In contrast, large parts of the red clusters of voxels in PC2 and PC3 matches the anatomy of the forceps minor and pyramidal tracts, and were associated with more severe forms of the disease, suggesting that they represent another WMH population.

Altogether, our results suggest that the whole burden of WMH in CADASIL results from the combination of at least 3 different regional WMH populations: one that progressively spreads with age while following the pattern of PC1, the evolution of which appears quite similar to that of WMH in sporadic forms of SVD. Another, mostly observed in severe patients, corresponding to further accumulation of WMH voxels in pyramidal tracts and forceps minor. Given its strong association with the volume of lacunes and brain atrophy, a potential candidate mechanism underlying this population might be secondary axonal degeneration. Finally, another WMH population, possibly specific to CADASIL, characterized by the accumulation of WMH voxels in anterior temporal poles and superior gyri, and which, for yet unknown reasons, are associated with milder forms of the disease. To note, the presence of fluid filled cavities embedded within the myelin sheath as observed in the CADASIL mouse model (17) and distinct from lesions usually observed post-mortem in sporadic SVD (18) might explain an increase of water content without significant myelin or axonal damage (4). Whether the presence of such myelin alterations could explain milder clinical severity in some patients remains however unknown.

Our study has several limitations. Whether patients having large extents of WMH in anterior temporal poles and superior frontal gyri actually develop with time milder forms of the disease will remain questionable until confirmation by further studies of long term follow-up data that are currently gathered. Also, the understanding of white matter damage from conventional MRI remains incomplete. Our approach relied on the delineation of WMH, but it is now widely admitted that WMH locally correspond to heterogeneous lesions and that white matter damage is also present in the so called normal appearing white matter (19). Similar approaches, not based on the study of a proxy such as WMH, but rather on the identification of quantitative MRI signatures of the different substrates of white matter damage will help delineating the local severity of white matter damage in patients suffering from SVD. Also, most of the variations of the pattern of WMH remain uncaptured by our algorithm. It is important to understand that given the strong reduction in the dimensionality of the problem when stepping from voxel analyses to principal component analyses, it was expected that a large part of the variability in WMH patterns would not be explained by this approach. In addition to this methodological explanation, whether the remaining unexplained variance is related to stochastic processes involved in the occurrence of white matter damage or to unidentified systematic sources of variations will require further studies.

While WMH were so far considered to have limited consequences in CADASIL (10), the results of the present study suggest that this view might have been misleading because different regional populations of WMH with potentially distinct mechanisms and clinical consequences were previously considered as a unique entity (6, 7, 10, 12). The presence of WMH in anterior temporal poles and superior frontal gyri might be associated with milder forms of the disease, independent of all other determinants of disease severity, for reasons that remain largely undetermined.

## Ethic statement

This study was carried out in accordance with the recommendations of the local IRB in Paris and Munich. All subjects gave written informed consent in accordance with the Declaration of Helsinki.

## Authors contributions

ED and FH: conception and design of the study, statistical analyses; FD, Mathieu D, J-FM, SR, and RS: conception and design of the study; Marco D, Martin D, and HC: conception and design of the study, acquisition and analyses of the data, manuscript corrections; EJ: conception and design of the study, acquisition and analyses of the data, drafting the manuscript.

### Conflict of interest statement

MarcoD reports honoraria for lectures from Bayer Vital GmbH and Pfizer Deutshland GmbH. The remaining authors declare that the research was conducted in the absence of any commercial or financial relationships that could be construed as a potential conflict of interest.
