# Supplementary material for: Different Types of White Matter Hyperintensities in CADASIL
Source: Front Neurol. 2018 Jul 10;9:526. doi: 10.3389/fneur.2018.00526 (PMC6048276; doi:10.3389/fneur.2018.00526)

Different types of white matter hyperintensities in CADASIL

Edouard Duchesnay, PhD^1^, Fouad Hadj-Selem, PhD^1^, François De Guio, PhD^2^, Mathieu Dubois, PhD^1^, Jean-François Mangin, PhD^1^, Marco Duering, MD^4^, Stefan Ropele, PhD^5^, Reinhold Schmidt, MD^5^, Martin Dichgans, MD^4^, Hugues Chabriat, MD, PhD^2,3^, Eric Jouvent, MD, PhD^2,3^

^1^ NeuroSpin, CEA, Paris-Saclay, Saclay, France; ^2^ Univ Paris Diderot, Sorbonne Paris Cité, UMR-S 1161 INSERM, F-75205 Paris, France; ^3^ AP-HP, Lariboisière Hosp, Department of Neurology, F-75475 Paris, France and DHU NeuroVasc Paris Sorbonne; ^4^ Institute for Stroke and Dementia Research, Klinikum der Universtät München, Ludwig-Maximilians- Universität LMU, Feodor-Lynen-Straße 17, D 81377 Munich, Germany; ^5^ Department of Neurology, Medical University of Graz, Austria;

**Corresponding author:** Pr Eric Jouvent, Service de Neurologie, Hôpital Lariboisière, 2 rue Ambroise Paré, 75010 Paris, France. Phone: 33 1 49 95 65 29, Fax: 33 1 49 95 25 96, email: eric.jouvent@aphp.fr

**Supplementary figure 1: theoretical background behind the spatially regularized principal component analysis approach**

**Supplementary Figure 1 legend:**

The principles of the spatially regularized principal component analysis approach used to determine the sources of variation in shape of white matter hyperintensities are explained with a practical example.

***
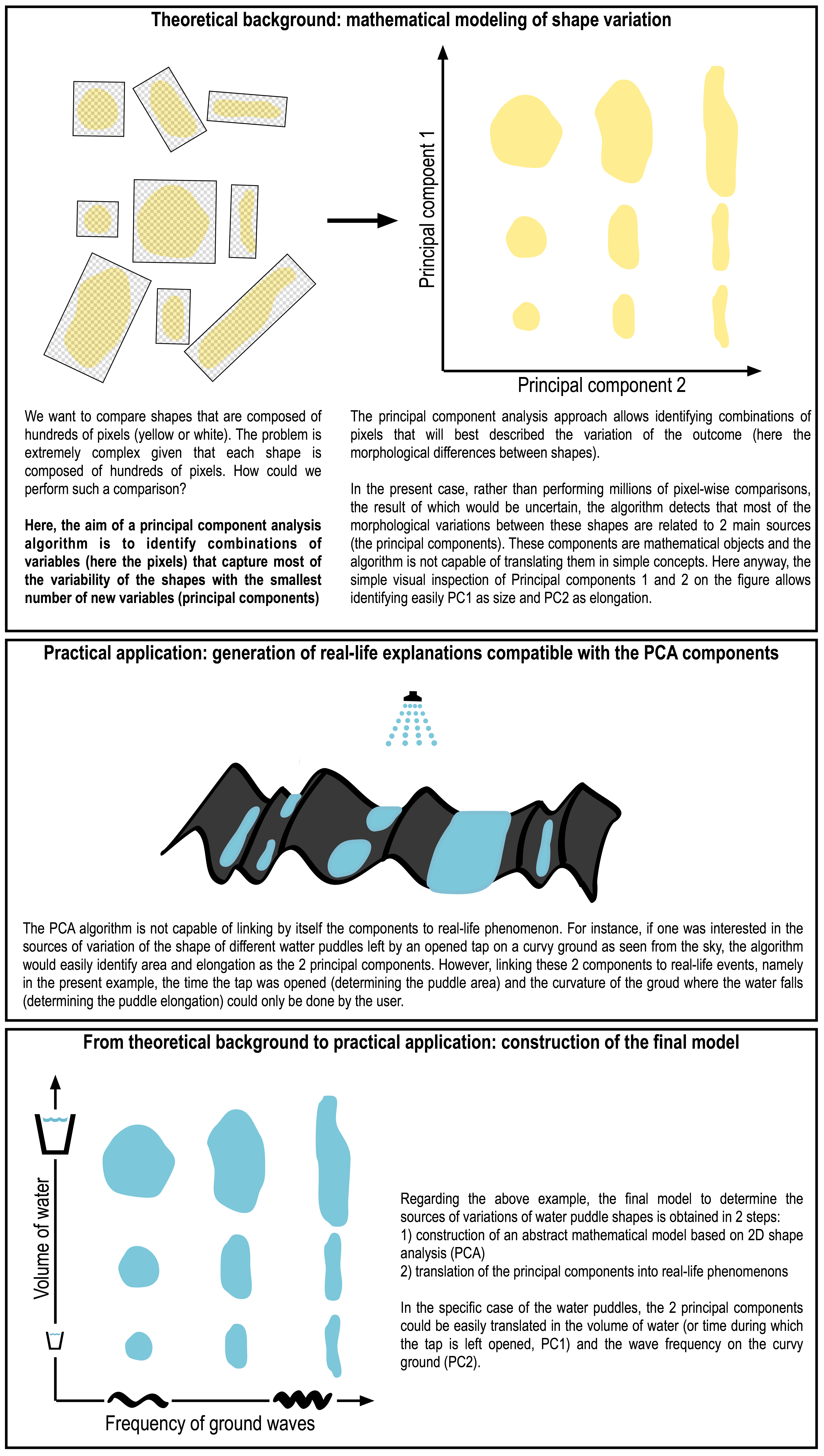
***

**Supplementary figure 2: clusters of voxels associated with poor outcomes in the third principal component overlaid on fibre bundles in the Johns Hopkins University atlas**

**Supplementary figure 2 legend:** The main clusters of voxels associated with both brain atrophy and more severe clinical outcomes in PC3 are overlaid on the main fibre bundles of the Johns Hopkins University Atlas. The figure clearly shows that the clusters are projected onto the pyramidal tract and the forceps minor.


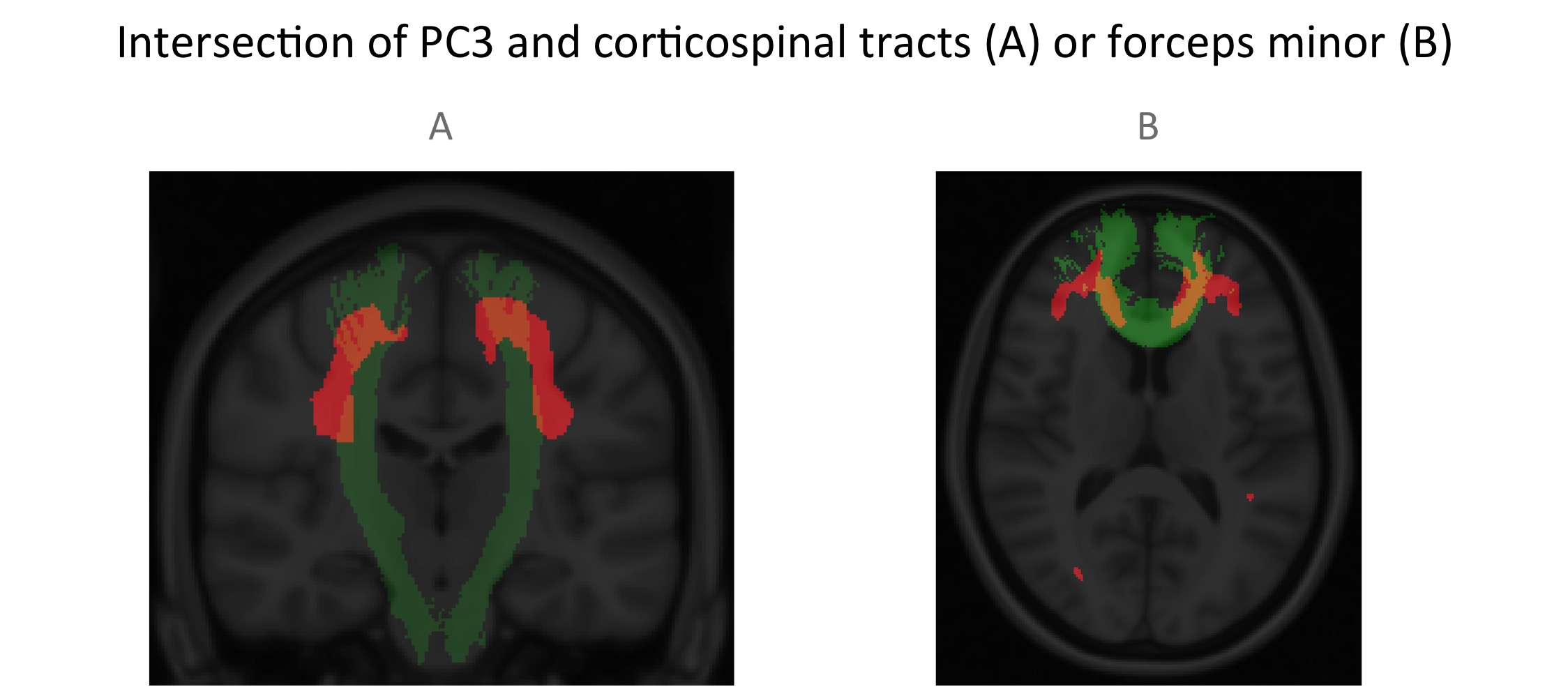

Supplement: Supplementary file 1 [file Data_Sheet_1.DOCX]
